# COVID‐19 and the uncertain future of HRM: Furlough, job retention and reform

**DOI:** 10.1111/1748-8583.12395

**Published:** 2021-07-04

**Authors:** Mark Stuart, David A. Spencer, Christopher J. McLachlan, Chris Forde

**Affiliations:** ^1^ Leeds University Business School University of Leeds Leeds UK; ^2^ Cranfield School of Management Cranfield UK

**Keywords:** Coronavirus Job Retention Scheme, furlough, job retention, labour hoarding, redundancy

## Abstract

The article argues that job retention should be a central aim and practice of human resource management (HRM). Set against the global COVID‐19 crisis, theoretical insights are drawn from strategic HRM planning and the economics of ‘labour hoarding’ to consider the potential benefits of workforce furloughing. Furlough has been supported in the UK by the Coronavirus Job Retention Scheme, which represents a novel, but *temporary*, state‐led shift from the UK's market‐orientated restructuring regime. We argue that the withdrawal of state‐financed furlough may mean a quick return in UK firms to the management of redundancy. Yet, if the crisis is to generate any benefit it must create the conditions for a more collaborative HRM that delivers for workers as well as business, with job retention as a core priority. While change in this direction will mean confronting deep‐rooted challenges—such as job security, good work and worker voice—such change remains vital in creating better and healthier workplaces.

**Abbreviations**
CIPD: Chartered Institute for Personnel and Development
CJRS: Coronavirus Job Retention Scheme
GFC: Global Financial Crisis
HPWS: high performance work systems
HRM: Human Resource Management
HMRC: Her Majesty's Revenue and Customs
OBR: Office for Budget Responsibility
OECD: Organisation for Economic Co‐operation and Development
ONS: Office for National Statistics
STC: short time compensation
STW: short time work
UK: United Kingdom

## 1 INTRODUCTION

This article argues that, in response to the COVID‐19 crisis, job retention should be seen as a central aim and practice of HRM, rather than simply an outcome variable. Governments in many countries have sought to protect those in work in the face of lockdown of economy and society through job retention schemes. According to the OECD, by May 2020 as many as 50 million jobs globally were being supported by job retention schemes, a tenfold increase compared to the Global Financial Crisis (GFC) of 2007/08 (Scarpetta et al., 2020). In the UK, 11.5 million jobs have been *furloughed* under the auspices of the Coronavirus Job Retention Scheme (CJRS) (ONS, 2021). Unlike schemes in many other countries, such as Germany and France, that extend and adapt existing provisions, the CJRS represents a novel departure from the typical, liberal market response to crisis and potentially challenges the dominant hard, ‘calculative’ approach to HRM adopted by many UK firms (Cregan et al., 2021). In contrast to previous crises in the UK, where employment fell in step with lower output, furloughing has helped to maintain people in jobs: firms have retained workers as opposed to making them redundant. Taking the UK case as a point of focus, the article argues that this ‘turn to job retention’ offers important insights for both the theory and practice of HRM.

**Practitioner Notes**

**What is known about the subject matter?**

- The COVID‐19 crisis presents a challenge, and potential opportunity for human resource management (HRM), with a large number of workers furloughed around the world.
- The UK government has sought to protect (up to 11.5 million) jobs through the crisis via the Coronavirus Job Retention Scheme (CJRS).
- The CJRS is a temporary intervention: when state‐support for furlough ends there is a danger that firms will quickly look to make workers redundant.
- Furlough is not well‐established in the UK compared to other countries, with the longer‐term practice of job retention constrained by challenges around job security, good work and worker voice.

**What does the article add?**

- The article makes the case for job retention as a key practice of HRM, rather than as an outcome.
- It details the context of the CJRS, explains how it represents a novel intervention in the UK's market‐based system of employment and situates it as a case comparatively.
- It considers the benefits of job retention through a novel synthesis of the literature on strategic HR planning and the economics of labour hoarding.
- The article argues the COVID‐19 crisis prompts cause for reflection on the renewal of HRM, with job retention as a central priority.

**Implications for practitioners**

- Job retention should be considered a core practice of HRM.
- As a practice of HRM, job retention should be seen as a central element of a wider investment‐based approach to people management that promotes job security, good work and worker voice.
- HRM practitioners should look to develop job retention strategies in partnership with wider stakeholders, as part of a renewal of HRM that looks to accommodate interests beyond immediate shareholder interests.

The UK case is instructive for two reasons. First, with no history of furloughing within UK firms, the CJRS offers insight into how furloughing can potentially shift expectations and norms around retention management. Secondly, as a paradigmatic example of ‘market‐led’ restructuring (Bergström, 2019; Gazier, 2008), the UK has a history of using redundancy as the default mechanism for dealing with crises. The introduction of furloughing is thus useful in showing how alternative responses to crisis can emerge, including those that mean firms hold on to workers in the face of crisis. Situating the UK case within a more comparative context, we use the example of furloughing to show how job retention can yield benefits to firms as well as workers.

In this context, we argue that the current crisis provides scholars with the opportunity to reflect on established HRM approaches as well as future directions for research. Specifically, we argue that an increased focus on job retention as a *practice* rather than an *outcome* of HRM shifts analysis away from orthodox perspectives on the management of redundancy and in doing so raises important questions about the reform of HRM through and beyond crisis. Our understanding of job retention as a practice is advanced through the novel integration of concepts drawn from the strategic HR planning and labour economics literatures. The theory of labour hoarding is useful in demonstrating how an active approach to retention management can reduce costs for firms while conferring benefits to workers. We further develop our theoretical contribution to the literature by framing our understanding of job retention as a core practice of HRM within the wider political economy of regimes of restructuring.

The article proceeds by discussing the links between restructuring regimes and varieties of furloughing, allowing us to situate the UK CJRS within a comparative context. We then synthesise theoretical insights from strategic HR planning and labour economics to illustrate how firms can reduce costs by adopting furloughing. The lesson is that firms can benefit from retaining labour and that short‐term cost‐cutting via redundancy can backfire economically. We add to HRM theory by showing how labour retention can be viewed as a HR practice, and that, like other HR practices, it can impact upon individual behaviour and organisational performance. Finally, we consider the longer‐term challenges for HRM, focusing on the UK, but drawing out the wider, comparative implications beyond this case. We are aware that the political and structural constraints on progressive reform remain acute, particularly given the market‐led regime of workforce adjustment and restructuring in the UK. But our argument is that lessons learned from the crisis should be used to remould the future direction of HRM. For us, any reform of HRM is best achieved through the creation of a new social contract—where job security, good work and worker voice are made central to the employment relationship. This argument applies as much to the UK as to other countries. At the heart of our argument is the idea that HRM can be reimagined and that this task becomes more urgent as state‐support for furloughing is withdrawn.

## 2 RESPONDING TO CRISIS: THE MOVE TO FURLOUGH AND JOB RETENTION

While COVID‐19 primarily emerged as a health crisis, it has nonetheless had considerable economic and labour market consequences. In this sense, it is useful to draw parallels with the response to previous economic crises. Bergström (2019) notes that economic crises bring into focus the effectiveness and consequences of specific policy measures aimed at avoiding job loss. Such measures are influenced by the approach to restructuring adopted within specific countries, with Gazier (2008) categorising ‘regimes of restructuring’ as market‐led, negotiated or state‐led. The traditional UK response has been market‐led and has emphasised wage and labour cost adjustment, with firms looking to shed labour and freeze or cut wages, within an institutional framework of limited employment protection (Lloyd, 1999). Under this regime, HRM during downturns in the UK has focused heavily on the management of redundancy. During the 2007/8 GFC, for example, redundancies soared from 4 per 1000 employees pre‐crisis to 12 per 1000 at its peak (ONS, 2020). State intervention was limited to job search programmes for the unemployed, with negligible public funds to support the labour market more generally (Bergström, 2019). The onus was on firms to manage their own way through crisis, which they did with a ‘general acceptability of managing redundancy’ (Lloyd, 1999: 181) and faith in their ability to recruit and train a new workforce during economic recovery.

In contrast to market‐led regimes of restructuring, negotiated or state‐led regimes offer alternative responses to crisis, through practices designed to support the management of retention rather than redundancy. The classic example would be short‐time work (STW) schemes, whereby firms benefit from a state subsidy to cover the costs of reducing workers' hours. Such schemes have been described as ‘win‐win’ initiatives for individuals, firms and states: the risk of redundancy for workers is mitigated; firms can retain workers' skills, and respond quicker to subsequent upturns by increasing hours; while governments can limit sharp spikes in unemployment (Pavlopolous & Chkalova, 2019). Such schemes are well‐established across European economies, and are typically celebrated for their positive effect on job retention (Arranz et al., 2019; Baleer et al., 2016; Brey & Hertweck, 2020). Germany, for example, managed to negotiate the GFC with only modest rises in unemployment, despite large falls in output.

Brey and Hertweck (2020) analysis of the impact of STW schemes in OECD countries during the GFC shows that such schemes were most beneficial for job retention where they were already well‐established. This may offer an important indicator of the long‐term potential of job retention schemes introduced in response to the COVID‐19 crisis. Scarpetta et al. (2020: 7) review of such schemes across the OECD during the pandemic found that ‘companies made massive use of job retention schemes that cut hours or put workers “on furlough”’, either through STW or wage subsidy schemes. In most cases (23 countries, two‐thirds of those studied), COVID‐19 job retention policies sought to amend and extend coverage of existing schemes. Notable examples include the long‐established German *Kurzarbeit* scheme, which was simplified to allow firms to claim up to 60%–80% of wage replacement costs (previously 67%) where 10% or more of the workforce have had their hours cut (previously 30%). At the peak of the first wave of COVID‐19 in May 2020, 19% of German workers were covered by the scheme compared to just 4% during the peak of the GFC (Scarpetta et al., 2020).

Likewise, in France, firms can claim against the Activité Partielle scheme, in place since 2013, but extended in response to COVID‐19 to provide employees with up to 70% of their gross wages (60% from October 2020) for up to 12 months (previously six) (Scarpetta et al., 2020). Take‐up was 33 times higher than during the GFC. The extension of the French scheme was negotiated between the state, unions and employers; an approach that also characterised a new STW scheme in Denmark. In the Danish case, a strong social model helped to shape a job retention scheme that covered between 75% and 90% of the wages of temporarily laid off workers (Muller & Schulten, 2020).

These examples highlight how established social settlements, and the presence of pre‐existing schemes for STW and retention have provided the foundations for many of the most extensive and wide‐ranging job retention responses during the pandemic. The take‐up of such schemes has been larger and more encompassing, in terms of workers' supported and sectoral coverage, than during the GFC (Scarpetta et al., 2020). The United States (US) is a notable exception. While furloughing is a more established response to downturns in the US (Lee & Sanders, 2013), Short‐Time Compensation (STC) programmes exist in only around half of all states, and despite enhanced government support (up to 100% wage replacement), take‐up during the crisis was negligible due to a lack of awareness by firms and the administrative burden of enrolment. Instead, furloughed workers had to rely on enhanced unemployment benefits (Scarpetta et al., 2020); unemployment rose from 3.8% to 13% in the 3 months to May 2020.

The UK offers an interesting point of comparison, with the traditional market‐led response replaced during the pandemic by a state‐led approach. Specifically, the UK government intervened to protect jobs and livelihoods via the CJRS. The product of initial ‘social partner’ dialogue with business and union groups, the CJRS represented a unique intervention within the UK labour market context. The CJRS allowed firms to furlough workers, from March 2020, with the state remitting 80% of an employee's wage, up to a maximum of £2500 per month. To qualify, an employee had to agree to be furloughed. A minimum of three weeks' furlough was required, and under the initial provisions of the scheme, furloughed workers were not allowed to do any productive work for their employer. Demand for the CJRS was high. HMRC data reports 1.3 million UK businesses accessed the scheme (cumulatively to March 2021), with 8.9 million jobs furloughed at the peak period in May 2020 (approximately a third of the UK labour force) (ONS, 2021).

The CJRS was revised several times. First, ‘flexible furlough’ was permitted from July 2020, to allow for reduced hours' working, and peaked at 1.55 million jobs in December 2020 (just under 40% of all furloughs) (ONS, 2021). Second, incremental employers' contributions were introduced from August 2020, but were subsequently dropped. Third, the scheme was extended a number of times, against a backdrop of concern amongst business and union leaders over its temporary nature, with a scheduled end‐date of September 2021.

The UK Chancellor's portrayal of the CJRS as an ‘unprecedented measure for unprecedented times’ implied it was among the most extensive and generous schemes in the world. A claim of exceptionalism that, as noted above, does not stand up to comparative scrutiny. Certainly, the UK has no history of state‐led, short‐time working or furlough support, and in this sense the CJRS can be seen as unprecedented in terms of domestic labour market policy. Comparatively, however, key questions emerge about how job retention in the UK can be fostered as a long‐term goal of public policy and HRM practice. What is distinctive about many continental European schemes, such as those in Germany, Denmark, Spain and France, is how they responded by strengthening *existing* ‘state‐led’ or ‘negotiated’ restructuring regimes (Bergström, 2019). These regimes are largely characterised by collective negotiations between the state, employer associations and trade unions through a mixture of institutionalised bipartite and tripartite bargaining (Gazier, 2008). Employment regulation reinforced by a social and firm‐level commitment to maintaining employment has meant there has been a stronger focus on sustaining jobs and maintaining training and investment in workers during downturns and crisis periods (Lloyd, 1999). This stands in contrast to the UK, where the shift in the regime of restructuring from market to state‐led was *prima facie* reactive, uncertain and time‐limited.

For our purposes, we are concerned with the implications of the CJRS for HRM. The practice of furlough, as noted, has no history in the UK, yet has been adopted as the primary measure of workforce adjustment in the context of COVID‐19. Evidence suggests that the CJRS, initially at least, helped to protect jobs and insulate workers from the threat of job loss. However, government prevarication in extending the scheme and uncertainty over its long‐term future negatively impacted the labour market: redundancies and unemployment increased from September 2020. This suggests that HR practice over job retention is extremely fragile, and the post‐September 2021 period highly uncertain. For those workers put on the CJRS, there remains the risk of redundancy. This raises an open question about how HRM may adapt beyond furloughing. Can schemes such as the CJRS help to generate conditions in which longer‐term, progressive change in HRM might occur? Reflecting on comparative experience, can a new social contract emerge from the crisis that focuses on sustaining the benefits that the CJRS has provided? In what follows, we examine arguments from both HRM and labour economics on the potential benefits of job retention and labour hoarding, which provide a theoretical basis for the development of job retention as a central practice of HRM.

## 3 MANAGING JOB RETENTION: INSIGHTS FROM STRATGEIC HR PLANNING AND THE ECONOMICS OF LABOUR HOARDING

HRM scholarship has paid limited attention to the retention of workers as a *practice*. There has been long‐standing interest in workforce adjustment strategies, with a particular focus on the management of layoffs (Cascio, 2010; McLachlan et al., 2021), but with minimal consideration of active organisational retention management. The high‐performance work systems (HPWS) literature sees labour retention (and conversely labour turnover) as an *outcome* of other HR practices, rather than something to be managed or adjusted on an ongoing basis and which might, itself, impact upon individual behaviour and organisational performance (Glebbeek & Bax, 2004; Wu et al., 2015). We contribute to the HRM literature by giving a more central focus to retention as a HR practice that can be strategized, implemented and actively managed within firms.

Older literatures on ‘manpower’ or workforce planning do hint at the potential for firms to develop and use active retention strategies to motivate staff and improve organisational performance. Used by many firms throughout the 1960s‐1980s, manpower planning fell out of favour from the 1990s, due to its perceived prescriptive and rigid nature (Reilly, 1996), with the process often reduced to a series of ‘how to’ steps, involving scanning the environment, planning scenarios, recruiting and training workers and developing workforce plans (Robinson & Hirsh, 2008). While this ‘cycle’ of activities pointed to something strategic, in practice it was often little more than a data gathering activity, aimed at generating evidence to feed into short‐term staffing plans (Sinclair, 2004). With the rise of strategic HRM in the 2000s, it might have been expected that the strategic management of retention would have become a more prominent concern; yet an anticipated ‘golden era’ of workforce planning (Sinclair, 2004) has not materialised, with the most recent CIPD guide (CIPD, 2018) on workforce planning highlighting that senior workplace leader's confidence in workforce planning has been eroded amidst volatile economic conditions.

Nevertheless, a case can be made for the management of retention on both economic and non‐economic grounds. This case adds considerable support to the CJRS. It also suggests that a focus on retention as a *practice* can help to reshape HRM, including in a progressive direction. For individual workers, practices focused on retention management are likely to have a positive impact upon discretionary effort, worker morale and job satisfaction. Recent work by Maley (2019) argues that investment in employee capabilities during periods of economic turbulence, including actively retaining staff and investing in training, can lead to improvements in individual motivation, commitment, productivity and longer‐term organisational performance. Studies of furloughing in other countries and contexts (notably the US, where workers are often placed on unpaid leave during crisis periods) reveal how workers perceive it as preferable to longer‐term wage cuts or redundancies (Baranik et al., 2019; Lee & Sanders, 2013), with firms undertaking furloughing seeing minimal reductions in satisfaction, and little negative effects on long‐term performance. In common with Maley (2019), these studies illustrate how both firms and workers can benefit from retention practices.

Analyses of the effects of workforce adjustment strategies alongside other HRM measures are also worth considering. Cregan et al. (2021) analysis of redundancies in the UK found that collaborative (‘soft’) HRM approaches, with an emphasis on fostering trust and commitment, helped to minimise negative reactions to redundancies, with employees' maintaining effort levels after layoffs. In contrast, calculative (‘hard’) HRM workplaces saw an immediate withdrawal of discretionary effort by remaining employees after layoffs; workers blamed management and perceived layoffs as an erosion of trust, and long‐term organisational performance was lower. Likewise, Cascio et al. (2021) study of 4000 American companies over the period 1990–2016 found that restructuring via employee downsizing offered a lower performance pay‐off in the longer‐term compared to retaining employees in downturns.

While not yet incorporated into the HRM literature, arguments about ‘hoarding’ in labour economics also support the idea of firms strategically retaining workers during downturns (see Biddle, 2014). These arguments focus on cost‐savings that accrue to firms from holding on to labour, which is seen as a ‘quasi‐fixed’ factor of production (Oi, 1962). Cost‐savings from labour hoarding are two‐fold. Firstly, there are lower costs of recruitment and training which result from retaining labour, as firms hold onto valuable firm‐specific skills, matching capital and organisational memory (Costas Dias et al., 2020). Firms can subsequently put ‘hoarded’ workers back to work, as output recovers, foregoing the need to recruit and train labour from the market. They can also avoid the costs of severance pay. While productivity may fall in downturns (as labour is retained and output falls), it can recover quickly in the upswing, as firms draw on labour they have retained (Bowers & Deaton, 1980).

Secondly, labour hoarding has an important psychological dimension. Specifically, it can help to maintain the morale of incumbent workers who would otherwise face unemployment. Evidence shows how unemployment has a ‘scarring’ effect on workers, and this effect can be long‐lasting and lead to lower well‐being and deteriorating health (Jahoda, 1982). Labour hoarding may reduce these problems, by connecting individual workers to their jobs in downturns (Dietz et al., 2010; Leitner & Stehrer, 2012). This is not to suggest that well‐being considerations disappear as a result of hoarding. Evidence from the COVID‐19 crisis suggests increased concern amongst employers about the mental health of their employees (Suff, 2020). Nonetheless, Burchell et al., (2020) note that, in the UK, the use of shorter working hours and furlough have provided some protection against any deterioration in mental health that is associated with unemployment: the mental health of furloughed workers in their study was equivalent to those in full‐time jobs and significantly better than workers made redundant. Under hoarding strategies, employers can also engage with and support workers prior to, and after, their return to work, which may have longer‐term benefits for their well‐being (and also productivity).

Support for the CJRS can be found, then, within both the HRM and labour economics literatures. The CJRS creates a hard financial incentive for firms to hoard labour. But, in its operation, it creates further benefits, as labour hoarding serves to reduce firm costs associated with recruiting and training once production begins again. The CJRS also allowed firms to furlough workers on a paid basis, helping to maintain a proportion of workers' income, in addition to their employment. Labour hoarding, encouraged by the CJRS, would seem likely to result in the maintenance, or even increase, of discretionary effort once workers resume employment, thus providing firms with further benefits.

However, the disincentives to hoarding come from the ‘market‐led’ regime of restructuring in the UK, which encourages a short‐term focus amongst firms, allows redundancies to take place with relative ease, and with little state support for maintaining employment, training and wider inter‐firm cooperation (Bergström, 2019; Lloyd, 1999). A more functional, ‘hard’ form of HRM has embedded itself in the UK as a result. The concern remains that when furloughing is withdrawn, firms will revert to a focus on managing redundancy—and any gains from furloughing will therefore be lost. Nonetheless, with its focus on job retention, the CJRS has shown that a different approach may be possible—an approach that could act as a platform for deeper reform of HRM more generally. The question is whether—and how—a different approach to HRM can be fostered after the CJRS is removed. We consider this question below, while also examining the broader implications of CJRS and job retention for the study of HRM.

## 4 CHALLENGES FOR HRM BEYOND THE COVID‐19 CRISIS: AN AGENDA FOR REFORM

With the CJRS and furlough in focus, our central contention is that the crisis affords an opportunity for reflection on the form and future direction of HRM, both in practice and theory. As a policy intervention, the CJRS has been regarded as a relative success (NIESR, 2020). It has arguably moderated levels of redundancy, held back a catastrophic increase in unemployment and opened up space for consideration of how job retention could become a core practice of HRM. Nevertheless, the support afforded by the CJRS is temporary. The danger is that the potential benefits derived from retaining workers through furloughing will disappear rapidly once firms begin to shed labour. More generally, any move to job retention as a practice of HRM confronts certain political and structural challenges that will need to be overcome—challenges that in the UK are linked to the extant regime of restructuring.

As others have argued, HRM has faced enduring problems of legitimacy and purpose. These problems were evident prior to the COVID‐19 crisis. Dundon and Rafferty (2018: 378–379), for example, argue that the function of HRM has been reduced to ‘minimising labour costs by implementing wage cuts, reducing employee pensions and benefits, or facilitating hostile takeovers and workforce restructuring’. For them, HRM has been captured by a pro‐market logic driven by the imperatives of financialisation and the increasing dominance of the shareholder value model. The consequences for workers have, inevitably, been detrimental. *Prima facie* the implications of a market‐led response to crisis (Bergström, 2019), characterised by a financialised concern for shareholder value, do not appear encouraging for an increased consideration of job retention.

Dundon and Rafferty's (2018) provocation reminds us of the need to locate the practice of HRM within a wider political economy approach (Vincent et al., 2020). Nonetheless, as Farndale et al. (2018, p. 466) note, even in the face of severe challenges, alternative directions are possible because ‘HRM works at the juncture of various essential corporate domains, giving it a comparative advantage to add value in times of crisis’. In this context, Dundon and Rafferty (2018) also make a case for a more ‘inclusive pro‐business’ approach to HRM, with a focus on business development and sustainability—centred around the fostering of mutual gains and worker wellbeing—that pays consideration not just to the financial gains of shareholders but also to the interests of employees and other business stakeholders. Arguably, an increased emphasis on job retention fits well with a reimagining of HRM around such a ‘pro‐business’ orientation. However, if the idea of retention as a *practice* is to become more embedded into the architecture of HRM, a number of key challenges need to be confronted. Below we address three challenges, namely job security, good work and voice at work. We then consider the scope for a renewal of HRM and the place of HRM scholarship in encouraging reform.

### 4.1 The challenge of job security

The most obvious threat from the crisis, beyond the period of furlough, is a severe decline in job security. Largely due to the perceived effectiveness of the CJRS in ‘subsidising labour hoarding’, the Office for Budget Responsibility revised its initial forecast of an increased unemployment rate of 11.9% by the fourth quarter of 2020 to 6.5% by fourth quarter 2021 (Francis‐Devine & Powell, 2021). Nonetheless, it is estimated that it will take 3 years to return to levels of economic activity reported in the quarter prior to the COVID‐19 lockdown (NIESR, 2020). Workers returning from furlough thus face heightened levels of uncertainty about their ongoing job security. Such uncertainty has been exacerbated by UK Government indecision over extensions to the scheme, which compares unfavourably to countries such as Germany and France that determined early in the crisis long‐term extensions to their respective schemes; in Germany until the end of 2021 and in France until 2023.

Furloughed workers have naturally been concerned about their ongoing job security. Real time survey data (Adams‐Prassl et al., 2020) found that furloughed workers were 15% more likely to fear losing their jobs before August 2020 than non‐furloughed workers. Likewise, a YouGov survey of employers found that half expected to lay‐off some furloughed workers following the withdrawal of CJRS (Smith, 2020). While the full extent of redundancy amongst furloughed workers will not become apparent until after the CJRS closes, there are grounds for concern. The UK Government's indecision over extending the CJRS (initially beyond October 2020) had a negative impact on the labour market: redundancies in the 3 months to November 2020 reached a record high of 395,000. While the unemployment rate has not risen sharply, wider signs of weakness in the UK labour market are evident. Notably, the claimant count increased by 115% between March and May 2020, while 738,000 fewer people were registered on payroll in the 12 months to April 2021 (Francis‐Devine & Powell, 2021). Estimates suggest that 9% of workers furloughed during the first lockdown were no longer in work by September 2020 (Brewer et al., 2020). These data suggest a labour market conducive to heightened job insecurity.

Labour market uncertainty in the context of the COVID‐19 crisis needs to be situated within the wider weaknesses of the UK's restructuring regime. The celebrated flexibility of the UK system means there are no direct responsibilities on employers to ensure security of employment (Rubery et al., 2016) and British workers have few social protections in one of the least regulated labour markets in the world. Employment security for many workers has become increasingly precarious, evident in the notable increase in the number of zero‐hours’ contracts (Alberti et al., 2018). A pessimistic reading would thus suggest that the CJRS offers little more than a ‘waiting room’ for redundancy. Importantly, no obligations have been placed on employers to retain furloughed staff beyond the CJRS. Ongoing responsibility for retention management has been left to employers. It is clear that job insecurity will become a more serious problem and that HRM will be required to adapt to this problem.

### 4.2 The challenge of good work

There is a recognised problem of good work—or lack of it—in the UK. The Taylor Review of Modern Working Practices highlighted this problem (Taylor et al., 2017). The rise of gig work and the platform economy has drawn attention to precarious work, insecure contracts and ‘bogus’ self‐employment, but the challenge of creating and sustaining good work is a much wider and longer‐standing problem that has intensified under financialised business models (Thompson, 2019). As Findlay and Thompson (2017) show, good work, the promotion of discretionary effort and worker well‐being, often placed at the centre of HPWS, mean little for many workers when faced with increased performance monitoring and management, more demanding expectations of effort and work intensity, and the blurring of work‐life boundaries. Likewise, work‐life balance and flexible working are increasingly seen as core HRM concerns and fit well with the good work agenda, but, as the Taylor Review argued, in practice a ‘one‐sided flexibility’ serving employers' interests tends to predominate (see also Rubery et al., 2016). Experiences of work have also become increasingly unequal—as labour market divisions have sharpened—depending on sectoral, skill and demographic differences.

Inequality at work is evident in workers' experiences of furlough. Younger workers and the low paid were far more likely to have been furloughed, whereas higher paid workers on furlough were more likely to have received salary top‐ups by their employers (Cominetti et al., 2020). It was more common for mothers to explicitly request furlough, raising questions about their longer‐term job security without adequate provisions for childcare (Adams‐Prassl et al., 2020).

Experiences of work intensity may also have carried over into furlough, even though the CJRS initially proscribed productive work. Evidence suggests that up to two‐thirds of workers did some work while on furlough. For Adams‐Prassl et al. (2020: 21), this suggests ‘the importance of worker autonomy in the decision to work whilst furloughed’. But it could equally be that furloughed workers felt the need to continue to work to safeguard their jobs: fear may have driven decisions. Moreover, 19% of furloughed workers were *expected* to work by their employers (Adams‐Prassl et al., 2020: 21). Fear of job loss may not be misplaced, as there is some evidence of employers using furlough as cover to restructure or replace jobs with technology (Gilbert et al., 2020).

As Gilbert et al. (2020) conclude, positive experiences of furlough are likely to be associated with employers that value and provide good work. While furloughing creates the basis for reciprocity between workers and employers, its capacity to generate and sustain good work will depend on the context in which it operates as well as its possible legacy effects. If, with the removal of the CJRS, HRM simply reverts to cost‐cutting and labour‐shedding the scope for good work will be diminished.

### 4.3 The challenge of voice

The CJRS was the product of dialogue between the state, capital and labour, but this did not extend to decisions on its operation, (re)design or withdrawal, unlike similar schemes in more negotiated regimes of restructuring, such as Denmark. Collective systems of voice have withered in the UK, with the decline of trade union membership and collective bargaining. Workplace decisions are increasingly the prerogative of management (Van Wanrooy et al., 2013). In place of collective representation and worker voice, HRM tends to stress the value of employee engagement, which is seen to contribute positively to job satisfaction, well‐being and discretionary effort. Yet, evidence suggest that in practice there is an ‘employee engagement deficit’, with surveys reporting that just a third of UK employees claim to be engaged at work (Findlay & Thompson, 2017; Rayton et al., 2012). Consequently, employee engagement is seen by many workers—in the absence of genuine voice mechanisms—as little more than a management‐driven agenda, offering little for workers in return.

The value of engagement with those on furlough would seem important for capturing the potential benefits of labour hoarding, but this demands meaningful mechanisms of voice. Such mechanisms might be fostered through two‐way communication, dialogue and engagement with trade unions and could focus on content issues such as the terms of furloughing and wider support structures, including investment in training during the furlough period. However, UK workers' rights to voice during furlough—as with restructuring and mass layoffs more generally—were minimal, compared to many other countries. Notionally, decisions over furlough were to be jointly agreed between employer and employee, but evidence shows that just 7% of workers volunteered for furlough, with ‘the vast majority of furlough choices… made by the employer’ (Gardiner & Slaughter, 2020: 5). In practice, employers retained control over the furloughing process. While there are some examples of tripartite dialogue over the end of furloughing in the UK, most notably the statement on Fair Work principles in Scotland (Scottish Government, 2020), in practice, employers have maintained control over the return to work and the concern is that, given the existing absence of effective voice mechanisms at work, this control will be used to shed labour once the CJRS ends.

### 4.4 The renewal of HRM

Job retention as a *practice* of HRM in essence signals a long‐term investment in human resources. Instead of simply responding to crisis by cutting labour costs, furlough offers the basis to build longer‐term, potentially more productive relations between an employer and its staff. In this regard, we argue that the CJRS offers a unique opportunity for HRM to be re‐oriented around longer‐term investment in people. In putting the focus on retention, however, the CJRS also puts models of HRM under the spotlight. In the market‐based context of the UK, this means opening up the practices that constitute the dominant, calculative approach to HRM to scrutiny (Cregan et al., 2021). We have explored above how the CJRS highlights challenges at the level of job security, good work and worker voice, and how these challenges present broader constraints on an investment‐focused HRM. These barriers need to be overcome if more progressive and inclusive forms of HRM are to be constructed in and beyond the COVID‐19 crisis. This, in turn, raises deeper questions around the political constraints that may inhibit any reform of HRM, as well as the contribution of HRM scholarship in challenging these constraints.

Despite its novelty in the UK context, the CJRS has promoted a *passive* rather than an *active* approach to labour retention—that is, companies have received support for their wage bill, without fundamentally reflecting on and changing their practice. If labour retention is to become more embedded as a practice beyond the crisis, it will need to do so against the institutionally impoverished context that characterises the UK's policy eco‐system. Specifically, the UK lacks the well‐developed structures of coordination and negotiation between government and employers' and workers' groups that have characterised the extension and adaptation of job retention schemes in countries such as Germany, Denmark, France and Sweden. In such countries, national policy formation is also supported by strong sectoral, regional and institutional configurations that foster social partnership and innovation between employers and employees and a longer‐term commitment to the idea of job retention.

Absent such collective decision‐making, the CJRS, for all its innovation and huge financial investment, evolved in a reactive, contingent and piecemeal fashion, largely divorced from wider labour market policy or long‐term planning. The UK government has been keen to stress the temporary nature of the CJRS, and its support for job retention has been disconnected from any wider consideration of HRM reform or its jobs recovery strategy post COVID‐19. Consequently, any longer‐term development of job retention as a practice of HRM beyond the support of the CJRS will remain the primary responsibility of employers, with little by way of the institutional ‘constraints’ that operate in more coordinated economies to influence and direct practice. This means that the opportunity for more progressive forms of HRM, moulded around a commitment to job retention, beyond the current crisis are highly uncertain.

The uncertainty of reform, we would argue, presents an important agenda, not just for organisations and HR practitioners, but also for researchers of HRM. Job retention, as we have highlighted above, has been a missing element in HRM research, both theoretically and empirically, which has tended to focus more on labour turnover as an outcome rather than seeing retention as an active practice of HRM. This neglect must be addressed if we are to more fully understand the dilemmas of HRM during the present crisis and beyond. More widely, the focus on job retention affords an opportunity to develop more expansive theoretical models of HRM that not only recognise and support wider practices of job retention in downturns, but also stress the value of greater social dialogue and partnership throughout the economic cycle.

Here retention of labour can be seen to encompass wider goals, such as supporting workers' ongoing development in work, and moving beyond conceptions of labour as a cost to be minimised. Models of HRM continue to pay little attention, either directly or indirectly, to labour retention. In HPWS approaches, labour retention rarely appears in ‘bundles’ or lists of HR practices, nor is it modelled as something that can be fostered through other practices, and that can be shaped through dialogue and engagement with stakeholders. Ironically, the UK's experience of the CJRS may offer some grounds for optimism, as there was strong support from both employers' groups and trade unions for the initiative. The task for HRM researchers, then, is draw out the potential benefits of job retention during crisis to promote a new post‐COVID‐19 social contract at work that may guide practitioners of HRM and harness and consolidate support amongst different stakeholders to secure more sustainable forms of social partnership. This social contract should put job security, good work and worker voice at the centre of HRM and create the basis for more democratic approaches to employment regulation whereby support for job retention is seen as a key element of any economic recovery.

## 5 CONCLUSION: BUSINESS AS USUAL OR ‘BUILD BACK BETTER’?

Using the example of the CJRS in the UK, we have shown how the COVID‐19 crisis could potentially renew both the theory and practice of HRM in a more progressive direction. By drawing conceptually from the strategic HR planning and labour economics literatures, the article argues that the turn to furloughing in the UK has highlighted the potential benefits to UK firms of retaining workers in downturns. It has also shown how, from the perspective of HRM research, there is a need to see job retention as a core *practice* of HRM. Therefore, a future theoretical focus for HRM researchers is to consider how firms might actively manage job retention, to the benefit of efficiency and employee well‐being.

The novelty of the CJRS within the UK restructuring regime stands in contrast to practice in many other countries that have sought to extend and adapt existing provisions, notably short‐time working schemes. While the level of state support for job retention across many economies in response to COVID‐19—which far exceeds anything offered during the GFC (Scarpetta et al., 2020)—raises an open question about the viability of such support in the longer‐term, even in more coordinated economies, it is possible to draw out some comparative lessons. First, in contrast to the CJRS, job retention schemes are more established in countries like Germany and France, and public policy initiatives are more embedded within labour market policies, institutional structures and organisation cultures that regard redundancy as a last resort. Second, while the CJRS was the product of an initial dialogue between government, employers' groups and unions, this dialogue does not compare to the level of social partnership that guides the design and implementation of similar schemes in countries like Germany, France, Sweden and Denmark. These institutional, regulatory and collaborative points of difference highlight more longer‐term and stable commitments to supporting job retention than in the UK.

The CJRS, then, has provided an opportunity for reflection on the value of job retention during crisis, even in a traditionally market‐based regime like the UK. However, as a temporary intervention, its benefits may be short‐lived, with the very real danger of ‘business as usual’ being restored following its withdrawal. Our central argument is to make the case for a different trajectory that places job retention at the heart of any economic recovery, with the renewal of HRM a key goal. This is no easy task. It will require wider systemic reform of the UK employment regime. Nonetheless, as the UK government looks to ‘build back better’ from the COVID‐19 crisis, and redefine itself in a post‐Brexit world, this is a critical moment for change. HRM has much to offer in advancing the case for change and can (and should) play a role in laying the foundations for a new social contract that places job security, good work and voice at the centre of employment relationships.

## CONFLICT OF INTEREST

None.

## Data Availability

Data sharing is not applicable to this article as no new data were created or analyzed in this study.
